# 75 Juvenile systemic sclerodermas: a case report

**DOI:** 10.1093/rheumatology/keac496.071

**Published:** 2022-10-07

**Authors:** A Remilaoui, H Hafirassou, A Ferhat, C Dahou-Makhloufi

**Affiliations:** Rheumatology Department of the Bab-El-Oued University Hospital, Algiers

## Abstract

**Background:**

Systemic scleroderma (SSc) is a rare generalized connective tissue disorder of poorly understood aetiology resulting in systemic fibrosis. It affects mainly women, usually in adulthood, but can occur at any age, although it is rare in children. Its prognosis is conditioned by cardiopulmonary involvement, which is the primary cause of mortality. Some innovative therapies have improved the management of this orphan disease.

We report an observation of a 14-year-old child with systemic scleroderma.

**Observation:**

Child B.Z, 14 years old, without any particular history, consulted for a Raynaud's phenomenon that had appeared 4 months earlier. Clinical examination reveals localized skin sclerosis on the face and extremities of the upper limbs (Rodnan score = 11), associated with inflammatory-type arthralgias. Biologically: SV = 38 mmH1, CRP = 87 mg/l, FAN at 1/320 and positive Anti-Scl70. Periungual capillaroscopy showed megacapillaries with altered capillary architecture. The frontal chest X-ray did not reveal any interstitial syndrome, the cardiac ultrasound was without abnormalities (no PAH). The diagnosis of SSc was retained, the patient was put on calcium channel blockers (Diltiazem at a dose of 120 mg/d) associated with dietary rules and NSAIDs on demand with good improvement.

**Conclusion:**

Very few cases of juvenile SSc have been reported in the literature. Its diagnosis should be based on the same ACR-EULAR 2013 criteria as those for adults. Its prognosis is conditioned by visceral damage which must be diagnosed and treated early.

